# Event-Related Potential Study of Recovery of Consciousness during Forced Awakening from Slow-Wave Sleep and Rapid Eye Movement Sleep

**DOI:** 10.3390/ijms231911785

**Published:** 2022-10-04

**Authors:** Krystsina Liaukovich, Sergei Sazhin, Pavel Bobrov, Yulia Ukraintseva

**Affiliations:** 1 Institute of Higher Nervous Activity and Neurophysiology of the Russian Academy of Sciences, 117485 Moscow, Russia; 2 Federal State Autonomous Educational Institution of Higher Education I.M. Sechenov First Moscow State Medical University of the Ministry of Health of the Russian Federation (Sechenov University), the N. V. Sklifosovsky Institute of clinical medicine, 119991 Moscow, Russia

**Keywords:** consciousness, awareness, awakening, ERP, REM, SWS, local-global paradigm

## Abstract

This work aimed to study the recovery of consciousness during forced awakening from slow-wave sleep (SWS) and rapid eye movement sleep (REM) in healthy volunteers. To track the changes in the degree of awareness of the stimuli during the transition to wakefulness, event-related potentials (ERPs) and motor responses (MR) in the auditory local-global paradigm were analyzed. The results show that during awakening from both SWS and REM, first, alpha-activity restores in the EEG, and only 20 and 25 s (for REM and SWS awakenings, respectively) after alpha onset MR to target stimuli recovers. During REM awakening, alpha-rhythm, MR, and conscious awareness of stimuli recover faster than during SWS awakening. Moreover, pre-attentive processing of local irregularities emerges earlier, even before alpha-rhythm onset, while during SWS awakening, the local effect we registered only after alpha restoration. The P300-like response both on global and local irregularities was found only when accurate MR was restored. Thus, the appearance in EEG predominating alpha-activity is insufficient either for conscious awareness of external stimuli or for generating MR to them. This work may help to understand the pathophysiology of sleep disorders well as conditions characterized by the dissociation between behavior and various aspects of consciousness.

## 1. Introduction

What is consciousness? What is it to be conscious? What are the neuronal correlates of consciousness? The exact answer to these questions has not yet been given, even though much research has been conducted [1,2]. There are many theories and definitions of consciousness [3]. Among neurophysiologists, consciousness usually refers to the waking state, as in the definition given by Zeman:

*“… consciousness is generally equated with the waking state, and the abilities to perceive, interact and communicate with the environment and with others in the integrated manner which wakefulness normally implies. Consciousness in this sense is a matter of degree: a range of conscious states extends from waking through sleep into coma”*

[3] (p. 1265)

This definition helps us to focus on another question—What is it to be conscious? Answering this question is particularly important in clinical surroundings concerning patients with disorders of consciousness that occur following coma. To respond to this question, clinicians often use the capacity to produce responses to commands [4]. Differentiating states of patients’ consciousness during recovery, clinicians introduced the term “level of consciousness” [5]. The level is considered one of the dimensions of consciousness, together with content (in a two-dimensional model, see [6,7]) or content and form (in a three-dimensional model [8]). The notion of a level of consciousness is also applicable to describe states during sleep or in the transition from sleep to wakefulness. It was shown that even when we sleep, our conscious life preserves [9]. I.e., after awakening from rapid eye movement sleep (REM), dreams with a complex plot can be recollected, and even after awakening from slow-wave sleep (SWS), the deepest stage of sleep, a person can remember some simple images [10]. However, only a low level of consciousness persists during sleep, characterized by a lack of awareness and command-following, and even after awakening, they do not recover immediately [11,12]. This makes sleep states particularly interesting for investigating the minimal neural mechanisms necessary and sufficient to maintain consciousness.

Several major theories try to explain the neuronal substrate of consciousness [13,14,15]. Nevertheless, there is no clear answer to the question of what mechanisms are sufficient for consciousness maintenance [16]; it is generally recognized that consciousness requires an integrated neural substrate [14]; in particular, the degree of cortico-cortical functional connectivity is important [17] and the EEG alpha-activity that provides this connectivity [18,19]. In addition, there is a universally accepted concept of alpha-rhythm as the rhythm of wakefulness [20]. Probably, alpha-activity is a necessary condition for the presence of a high level of consciousness. However, is it sufficient?

One approach is investigating consciousness in the dynamic or its transition states—in the recovery, for example. The awakening of a comatose patient is difficult to predict, so assessment of neuronal correlates of the recovery of consciousness from coma is rarely available. Thus, the most appropriate model for studying transitional states of consciousness seems to be that of awakening from sleep. High levels of consciousness—full awareness and responsiveness—are absent during sleep, possibly because the brain’s capacity for information integration is restricted [21]. During non-rapid eye movement sleep (NREM), cortical neurons become bistable and alternate between a depolarized active up-state and hyperpolarized silent down-states [22]. This bistability prevents the emergence of sustained, complex thalamocortical interactions; therefore, a cortical response to stimulation becomes more local and stereotypical [21,23]. During REM, when the EEG is desynchronized, a cortical response is more widespread, but functional connectivity differs from those in the waking state [21,24]. The differences in functional connectivity and neuromodulation lead to differences in the perception of external stimuli in these states. So, during REM, the awakening threshold is as high as in SWS, but significant stimuli can cause faster wakening than from SWS [25,26], and a person is more alert when aroused from REM than from NREM [11]. However, even REM awakening is not immediate. Usually, there is a gap between an EEG-awakening (i.e., alpha appearance) and behavioral awakening (the sleeper’s own acknowledgment of waking) [11,12]. Why is a person not responding during this period? Possibly the conscious awareness already persists, but “it is difficult to organize a response” [11].

How can we probe consciousness during awakening? This includes the first seconds, when the capacity of command-following is not yet restored. One possible measure of conscious awareness is based on evaluating cerebral responses to violations of temporal regularities that are either local in time or global across several seconds. It is implemented in the local-global paradigm (LGP), which was invented as a tool to differentiate the complexity of conscious processing of external information in patients with disorders of consciousness [27,28]. As our brain is constantly generating and updating a mental model of the environment [29], LGP, using violations of two types of regularities, aims to dissociate two hierarchical levels of auditory predictive coding. The violation of local regularity, i.e., the appearance of an unexpected deviant sound after many identical sounds, reflects a disruption at the short-time scale. In addition, violation of global regularity, i.e., the appearance among repeating groups of five sounds with the same pattern of one group with an unexpected pattern, reflects a long-time scale disruption [27].

In EEG, in response to a violation of local and global regularities, different event-related potentials (ERPs) are registered: MMN and P3a component and P300 (or P3b component), respectively. MMN (MMN/P3a complex) is more attention independent—pre-attentive; therefore, it is registered in conscious, conscious but distracted [30], and even unconscious states in patients [27,28]. P300, or P3b, demands activation of higher-order areas, i.e., broad cortico-cortical activation of the frontal-parietal network associated with attention and working memory [27,31]. Therefore, responses to global deviant trials are registered only when a participant is aware of the violation of global regularity [27,32]. P300 is also considered as a neural response to stimulus significance [33,34]; therefore, it is not registered in an unconscious state [27,35] or in a wake but attentionally distracted state [31]. Thus, the global effect or the appearance of P300 (or P3b) might be considered as a signature of the conscious processing of auditory regularities. During sleep, our processing of external stimuli is disrupted, and only automatic change detection persists to some degree [36]. Specifically, in response to LGP, a local mismatch response was registered across NREM1, NREM2, and REM but with an incomplete structure, and neither the classical MMN/P3a complex nor P300 was detected [32]. During SWS, most researchers failed to find any response, even on local irregularities [36,37]. However, some questions are still not answered: At which point in the continuum of the transition from sleep to wakefulness emerges the processing of global irregularities? In addition, at which point do conscious reactions to local and global violations reappear? In addition, what are the differences in its recovery during SWS and REM awakenings? We suggest that using LGP, we can track the change in the degree of conscious awareness of the stimuli on gradual steps of state transitions. Learning more about how we regain consciousness during awakening from sleep may help find reliable predictors of the recovery of comatose patients. A comparison of progressive steps of SWS-wake and REM-wake transitions will lead to a better understanding of insomnia mechanisms and expand the range of diagnostic options.

Our research aimed to study the neural correlates that underlie the recovery of a high level of consciousness—our awareness of external stimuli and timely response to these stimuli—during forced awakening from SWS and REM. By registering ERPs and motor response (MR), we aimed to answer the following questions: (a) at which moment of the transition from sleep to wakefulness a MR to target stimuli recovers; (b) does this moment coincide with the appearance of the alpha-rhythm; (c) at which moment of transition from sleep to wakefulness two hierarchical levels of auditory predictive coding emerge: low-level pre-attentive response to a violation of local regularity and high-level conscious response to a violation of global regularity; (d) what are the differences in recovery of conscious awareness of stimuli during SWS and REM awakenings.

## 2. Results

### 2.1. Polysomnographic Data

The polysomnographic data were analyzed according to the awakening session: separately for the nights with awakenings from SWS and for the nights with those from REM (Appendix A, Table A1). No difference in sleep architecture between the two sessions was found.

### 2.2. Criteria for Division of Forced Awakenings from SWS and from REM on Consecutive Steps

The recordings were visually scored. First, we looked for the moment of MR recovery. In the recordings, we marked two consecutive time points: the moment of first MR, albeit it was false or slowed down (reaction time exceeded 550 ms), and the moment when MR became accurate and timely (reaction time is from 150 to 550 ms). Additionally, we marked the time point of occipital alpha-rhythm restoration in EEG, i.e., when alpha-activity became predominating.

**Figure 1:**
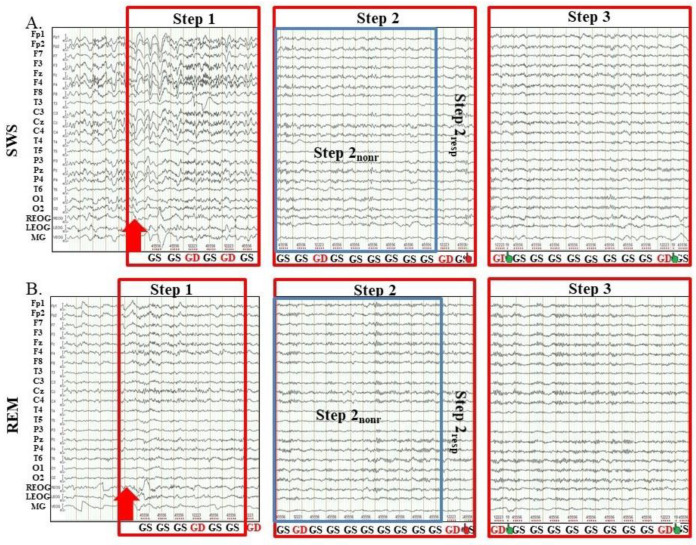
Examples of Steps of awakenings in the sessions with awakening from slow-wave sleep and rapid eye movement sleep. SWS, slow-wave sleep; REM, rapid eye movement sleep; Step 1, period of awakening when delta or theta prevail in EEG, the motor response is absent; Step 2, period of awakening when alpha prevails in EEG, the motor response is either absent or slow; Step 2_nonr_, period of awakening when alpha prevails in EEG, the motor response is absent; Step 2_resp_, period of awakening when alpha prevails in EEG, the motor response is slow; Step 3, period of awakening when alpha prevails in EEG, the motor response is accurate and timely; red arrow, the start of alarm sound; GS, global standard; GD, global deviant; the hand, motor response; the red hand, the motor response is slow; the green hand, the motor response is accurate and timely.

Even though SWS and REM differ in neuromodulation and arousal [38], there are some common patterns of awakening from them. In particular, both during awakening from SWS and REM, first, alpha-activity restored in the EEG, and only then the MR to target stimuli recovered. Therefore, we divided the awakenings, both from SWS and REM, into three Steps: (1) either delta or theta waves prevail in EEG; (2) alpha-rhythm recovered and prevail in EEG; MR is absent or slowed down; (3) MR is comparable to that in a waking state before sleep (examples are shown in Figure 1).

### 2.3. Comparison of EEG and Behavioral Characteristics of Forced Awakenings from SWS and REM

#### 2.3.1. Types of Awakenings from Sleep

There were different types of awakenings, both from SWS and REM. There were cases when no awakenings were registered, i.e., the participants did not wake up to the alarm and continued sleeping all the time when the sounds were presented. Additionally, there were partial awakenings during which alpha-activity recovered; however, the participants stayed at Step 2, i.e., their MR was false or delayed and did not become accurate and timely. During some awakenings, after reaching Step 2, the participants dozed off for some seconds (they stopped responding to target stimuli, and NREM1 was registered) and then woke up by themselves and restarted responding. In addition, such fluctuating between Step 2 and Step 1 sometimes lasted up to 10 s before the participant finally reached Step 3 and responded to target stimuli accurately and timely. In addition, lastly, there were awakenings when consecutive steps followed one after the other, and the participants reached Step 3 without returning to sleep or previous steps.

The number of cases with partial awakenings did not differ between sessions. The percentage of no awakenings was higher when participants were woken up from SWS, and the percentage of full awakening was higher when participants were woken up from REM (*p* = 0.023 and *p* = 0.001, respectively, Appendix B, Table A2).

We excluded from further analysis trials when the participants did not wake up at all, i.e., when EEG awakening was not registered. In partial awakenings and in awakenings, which were interrupted by falling asleep (when NREM2 was registered), we analyzed EEG, reaction time, and ERP data only on the first Steps. Moreover, we excluded from the analysis one awakening with excessive muscle activity.

#### 2.3.2. Difference in Latency of Each Step of Awakening

A comparison of awakenings from SWS and REM showed that at Step 1, they differed in EEG activity: after awakening from SWS, high amplitude delta rhythm predominated in EEG, while after awakening from REM, theta activity prevailed (Figure 1). At Step 2 and Step 3, EEG was comparable in both sessions, and there was only a difference in the timing of Steps: during the transition from REM to wakefulness, both the recovery of the alpha-rhythm and the restoration of the motor response came earlier. Further, to compare the speed of SWS and REM awakenings, we calculated the latencies of steps of only full awakenings when the participant reached Step 3. In addition, we excluded from analysis awakenings which contained returning to sleep or previous steps, and in some awakenings, we excluded steps with an excessive movement activity. So, here we present the timing of awakenings when their consecutive steps followed one after another, keeping in mind that usually awakening is not so consistent and dogmatic and lasts longer. Obtained results show that during awakening from SWS, the first burst of occipital alpha-rhythm was recorded not earlier than 15.24 ± 19.82 s after the alarm, while during awakening from REM—after 2.42 ± 2.35 s (*p* < 0.001, Table 1). Similarly, the first MR, albeit delayed, during awakening from SWS was recorded later than during awakening from REM (21.52 ± 13.55 s and 8.41 ± 7.05 s, *p* < 0.001, respectively, Table 1). According to the appearance of the first delayed MR, we divided Step 2 into two sub-steps: Step 2 nonresponsive (Step 2_nonr_) and Step 2 responsive (Step 2_resp_). The first correct and timely motor response, which we considered the beginning of Step 3, also came later during SWS awakening than during awakening from REM (40.22 ± 20.87 s and 22.72 ± 14.99 s, *p* < 0.001, respectively, Table 1).

#### 2.3.3. Difference in Latency of MR Recovery on Local and Global Irregularities

Next, we compared the latencies of Step 2 and Step 3 and RTs for sound sequences containing local or global irregularities separately. Local irregularity is a short-time scale violation (the appearance of one deviant sound), and it is easier to detect than global or long-time scale irregularity (the appearance of a deviant pattern of sounds) even at low arousal levels [32]. The RT analysis showed that responses to local irregularities in most states (Wake_pre_ and Wake_post_, and Step 3 of awakening from SWS and REM) were significantly faster than to global irregularities. Only at Step 2_resp_, there were no significant differences, possibly because performance at this Step, in general, was very unstable (Table 2). We also expected a faster recovery of MR on local irregularity. However, when we analyzed Step 2_resp_ and Step 3 latencies in different sessions separately and even in SWS and REM sessions together, we did not find significant differences between local and global irregularities (Table 2).

#### 2.3.4. Circadian Factor

Analysis of circadian timing of awakenings from SWS and REM showed more awakenings from SWS during the first part of the night and, on the contrary, more awakenings from REM during the last part of the night (62.3 % and 37.7 % for SWS awakenings and 34.9 % and 65.1 % for REM awakenings, respectively, *p* < 0.001, Appendix C, Table A3).

### 2.4. Event-Related Potentials Study

A further question we explored was at which step of awakening emerges auditory predictive coding. When do pre-attentive responses on local irregularity recover and when does awareness of the violation of global regularity occur? Do brain responses occur earlier than motor responses? In addition, were there any differences between SWS and REM awakenings in the ERPs in response to irregularities?

#### 2.4.1. Event-Related Potentials during Wakefulness

First, we analyzed the ERPs on target irregularities in the waking state before and after the night’s sleep. Using the cluster-based permutation test, we compared ERPs during Wake_pre_ and Wake_post_ in the sessions with awakening from SWS and REM. No difference in ERPs between the two awakening sessions was found. Since the sleepiness (VASS and SSS) scores also did not differ between sessions, we pooled the ERP data from the two sessions and analyzed the united Wake_pre_ and Wake_post_ data. For evening and morning VASS and SSS data in two awakening sessions, see Appendix D, Table A4.

##### Local Irregularities

**Figure 2:**
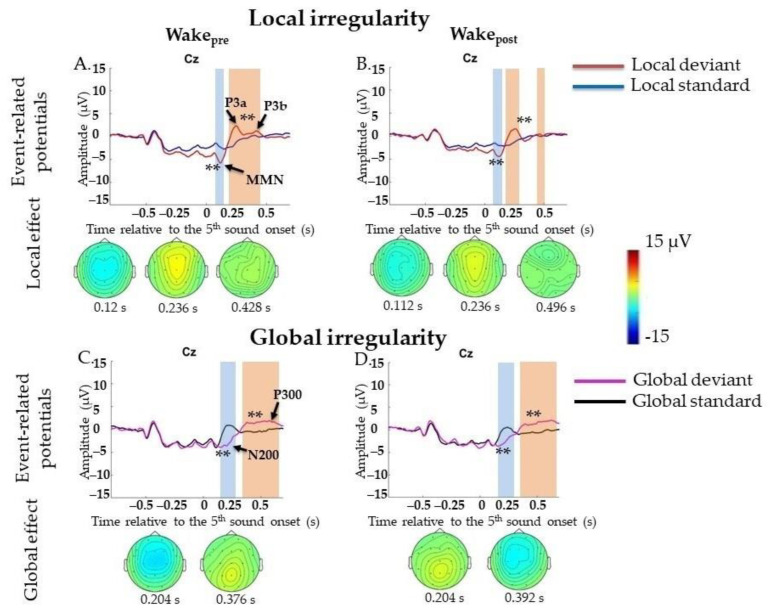
Event-related potentials (ERPs) to violation of local and global regularities in the waking state before (Wake_pre_) and after (Wake_post_) sleep. Wake_pre_, performance before sleep; Wake_post_, performance after sleep; s, second; red line, averaged local deviants; blue line, averaged local standards; magenta line, averaged global deviants; black line, averaged global standards; local effect, local deviants minus local standards; global effect, global deviants minus global standards. (**A**,**B**) Local mismatch response in EEG in Wake_pre_ session and Wake_post_ session. (**C**,**D**) Global mismatch response in EEG in Wake_pre_ session and Wake_post_ session. The blue-shaded region is a pool of negative clusters. The orange-shaded region is a pool of positive clusters. ** *p* < 0.01. Topographies of either local or global effect are shown for the maximum of ERP peak amplitude.

ERPs to local irregularities were analyzed by comparing responses to LD and LS as a contrast termed the local effect (LD-LS). In the Wake_pre_ session (6131 standard segments and 2536 deviant segments), a significant local effect was found for frontocentral MMN (0.0–0.148 s, *p* = 0.008), followed by the P3a/P3b complex (0.16–0.696 s, *p* = 0.002) (Figure 2A). Broad positivity in the 0.164–0.696 s window can be visually divided into two peaks: frontocentral positivity (P3a) (0.16–0.264 s) with its maximum amplitude peak at approximately 0.232 s at the Cz electrode and later centroparietal (P3b) at approximately 0.428 s at the C3 and C4 electrodes. All results were replicated in Two-Way ANOVA (Appendix E, Table A5). Importantly, the factor “participant” should be kept in mind (Appendix E, Table A5).

In the Wake_post_ session (5149 standard segments and 2095 deviant segments), a similar result for local effects was found: MMN (0.0–0.152 s, *p* = 0.006), which was followed by broad positive complex P3a/P3b (0.16–0.696 s, *p* = 0.002) (Figure 2B). All results were replicated in Two-Way ANOVA (Appendix E, Table A5). Importantly, the factor “participant” should be kept in mind (Appendix E, Table A5).

##### Global Irregularities

ERPs to violations of global regularities were analyzed by comparing responses to GD and GS. In the Wake_pre_ session (5692 standard segments and 2445 deviant segments), a significant global effect (GD-GS) was found for N200 (0.136–0.316 s, *p* = 0.002), followed by broad centroparietal P300 (0.26–0.696 s, *p* = 0.002) (Figure 2C). All results were replicated in Two-Way ANOVA (Appendix F, Table A6). Importantly, the factor “participant” should be kept in mind (Appendix F, Table A6).

In the Wake_post_ session (5080 standard segments and 2046 deviant segments), a similar result for the global effect was found: N200 (0.14–0.332 s, *p* = 0.006), which was followed by broad centroparietal P300 (0.256–0.696 s, *p* = 0.002) (Figure 2D). All results were replicated in Two-Way ANOVA (Appendix F, Table A6). Importantly, the factor “participant” should be kept in mind (Appendix F, Table A6).

#### 2.4.2. Event-Related Potentials during Awakenings from SWS

##### Local Irregularities

**Figure 3:**
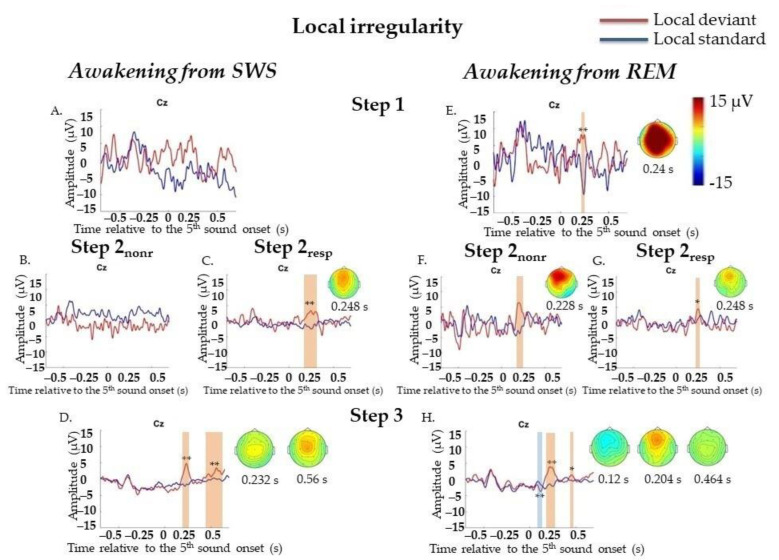
Event-related responses to violation of local regularity in the sessions with awakening from slow-wave sleep and rapid eye movement sleep. SWS, slow-wave sleep; REM, rapid eye movement sleep; Step 1, period of awakening when delta or theta prevail in EEG, the motor response is absent; Step 2_nonr_, period of awakening when alpha prevails in EEG, the motor response is absent; Step 2_resp_, period of awakening when alpha prevails in EEG, the motor response is slow; Step 3, period of awakening when alpha prevails in EEG, the motor response is accurate and timely; s, second; red line, averaged local deviants; blue line, averaged local standards; local effect, local deviants minus local standards. The sessions with awakening from SWS for all Steps separately (**A**–**D**). The sessions with awakening from REM for all Steps separately (**E**–**H**). The blue-shaded region is a pool of negative clusters. The orange-shaded region is a pool of positive clusters. * *p* < 0.025, ** *p* < 0.01. Topographies of local effect are shown for the maximum of ERP peak amplitude. The data, which were not replicated in Two-way ANOVA, are not marked with asterisks.

We did not find significant differences between LD and LS responses at Step 1 (72 standard segments and 42 deviant segments). At Step 2 (558 standard segments and 161 deviant segments), we found only frontocentral positivity (P3a) in a 0.216–0.268 s window, *p* = 0.014. Since we expected that awareness of stimulus significance could be restored earlier than the ability to generate an accurate and timely MR, we divided all trials on Step 2 according to the presence of MR and analyzed them separately. However, at Step 2_resp_ (455 standard segments and 132 deviant segments), again, only P3a was detected (0.208–0.312 s, *p* = 0.006), and we did not find any P3b-like response. At Step 2_nonr_ (103 standard segments and 29 deviant segments), no significant responses on LD were found. At Step 3 (799 standard segments and 314 deviant segments), ERPs on LD were similar to those in the awake state: the later P3a/P3b complex could be divided into two peaks: frontocentral P3a (0.176–0.288 s, *p* = 0.002) and later P3b (0.408–0.68 s, *p* = 0.002) (Figure 3A–D; data for Step 2 are not shown). All results were replicated in Two-Way ANOVA (Appendix E, Table A5).

##### Global Irregularities

**Figure 4:**
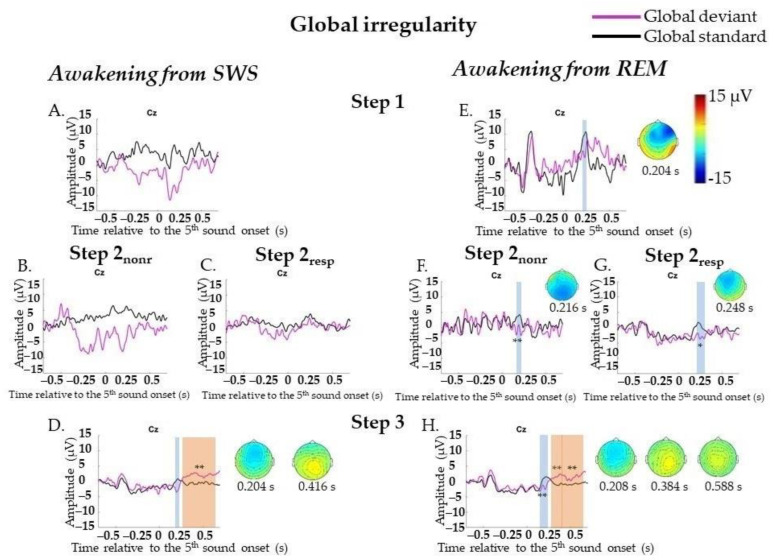
Event-related responses to violation of global regularity in the sessions with awakening from slow-wave sleep and rapid eye movement sleep. SWS, slow-wave sleep; REM, rapid eye movement sleep; Step 1, period of awakening when delta or theta prevail in EEG, the motor response is absent; Step 2_nonr_, period of awakening when alpha prevails in EEG, the motor response is absent; Step 2_resp_, period of awakening when alpha prevails in EEG, the motor response is slow; Step 3, period of awakening when alpha prevails in EEG, the motor response is accurate and timely; s, second; magenta line, averaged global deviants; black line, averaged global standards; global effect, global deviants minus global standards. (**A**–**D**) The sessions with awakening from SWS for all Steps separately. (**E**–**H**) The sessions with awakening from REM for all Steps separately. The blue-shaded region is a pool of negative clusters. The orange-shaded region is a pool of positive clusters. * *p* < 0.025, ** *p* < 0.01. Topographies of global effect are shown for the maximum of ERP peak amplitude. The data, which were not replicated in Two-way ANOVA, are not marked with asterisks.

No statistically significant ERPs on GD were found at Step 1 (164 standard segments and 77 deviant segments) or Step 2 (322 standard segments and 115 deviant segments), not even when analyzing Step 2_nonr_ (109 standard segments and 47 deviant segments) and Step 2_resp_ (213 standard segments and 68 deviant segments) separately. N200 (0.164–0.236 s, *p* = 0.02) and later broad posterior positivity (0.296–0.696 s, *p* = 0.002) were found only at Step 3 (760 standard segments and 297 deviant segments). A broad posterior positivity can be visually divided into two peaks: posterior positivity with its maximum amplitude peak at approximately 0.416 s at the Pz electrode and later bilateral central positivity at approximately 0.58 s at the Cz electrode (Figure 4A–D; data for Step 2 are not shown). The result for N200 was not replicated, and the result for P300 was replicated in Two-Way ANOVA (Appendix F, Table A6). Importantly, the factor “participant” should be kept in mind for P300 at Step 3 (Appendix F, Table A6).

##### Target Irregularities Versus Nontarget Irregularities

To find out at which step emerged the difference in evoked responses on target and nontarget deviants, we compared ERPs on the trials containing the same sounds from different awakenings—where the analyzed trial was the target and where it was nontarget: LD vs. GS and GD vs. LS.

**Figure 5:**
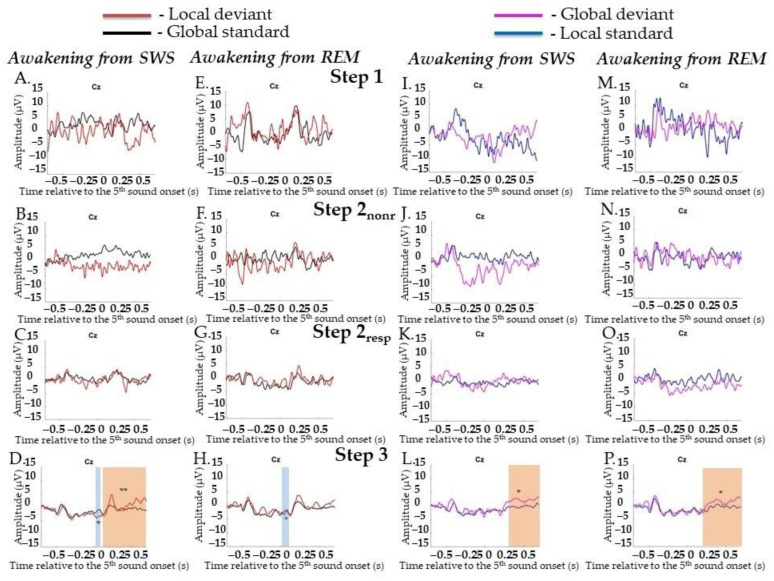
Event-related responses to violation of local regularity and global regularity in the sessions with awakening from slow-wave sleep and rapid eye movement sleep. SWS, slow-wave sleep; REM, rapid eye movement sleep; Step 1, period of awakening when delta or theta prevail in EEG, the motor response is absent; Step 2_nonr_, period of awakening when alpha prevails in EEG, the motor response is absent; Step 2_resp_, period of awakening when alpha prevails in EEG, the motor response is slow; Step 3, period of awakening when alpha prevails in EEG, the motor response is accurate and timely; s, second; red line, averaged local deviants; black line, averaged global standards; magenta line, averaged global deviants; blue line, averaged local standards. (**A**–**D**) The session with awakening from SWS for local deviants and global standards. (**E**–**H**) The session with awakening from REM for local deviants and global standards. (**I**–**L**) The session with awakening from SWS for global deviants and local standards. (**M**–**P**) The session with awakening from REM for global deviants and local standards. The blue-shaded region is a pool of negative clusters. The orange-shaded region is a pool of positive clusters. * *p* < 0.025, ** *p* < 0.01.

Neither LD and GS nor GD and LS differed at Step 1 and Step 2. When we analyzed Step 2_nonr_ and Step 2_resp_ separately, we did not find any differences also. At Step 3, a difference between LD and GS was found for MMN (0.0–0.144 s, *p* = 0.018) and the P3a/P3b complex (0.144–0.696 s, *p* = 0.002) (Figure 5A–D; data for Step 2 are not shown). A difference between GD and LS was found for P300 (0.4–0.696 s, *p* = 0.022) (Figure 5I–L, data for Step 2 are not shown). No statistically significant difference in early effects between pairs was found.

#### 2.4.3. Event-Related Potentials during Awakenings from REM

##### Local Irregularities

At Step 1 (32 standard segments and 19 deviant segments) and Step 2 (290 standard segments and 97 deviant segments), P3a (0.216–0.26 s, *p* = 0.01 and 0.184–0.276 s, *p* = 0.004, respectively) was found. When we analyzed Step 2_nonr_ (65 standard segments and 29 deviant segments) and Step 2_resp_ (225 standard segments and 68 deviant segments) separately, P3a was found for each sub-step (0.176–0.26 s, *p* = 0.01 and 0.204–0.272 s, *p* = 0.018, respectively). At Step 3 (911 standard segments and 331 deviant segments), MMN (0.012–0.132 s, *p* = 0.008) and later P3a (0.156–0.272 s, *p* = 0.006) and P3b (0.432–0.476 s, *p* = 0.018) were found (Figure 3E–H, data for Step 2 are not shown). All results were replicated, except the P3a result at Step 2_nonr_, in Two-Way ANOVA (Appendix H, Table A5). Importantly, the factor of the participant should be kept in mind (Appendix H, Table A5).

##### Global Irregularities

At Step 1 (157 standard segments and 71 deviant segments) and Step 2 (349 standard segments and 128 deviant segments), N200 (0.172–0.228 s, *p* = 0.042 and 0.156–0.288 s, *p* = 0.006, respectively) was found. Analyzing Step 2_nonr_ (46 standard segments and 16 deviant segments) and Step 2_resp_ (303 standard segments and 112 deviant segments) separately, N200 was found for each sub-step (0.208–0.224 s, *p* = 0.008 and 0.16–0.292 s, *p* = 0.012, respectively). At Step 3 (846 standard segments and 302 deviant segments), N200 with more central topography (0.136–0.248 s, *p* = 0.002) and later P300 (0.252–0.492 s, *p* = 0.002 and 0.496–0.696 s, *p* = 0.002) were found (Figure 4E–H, data for Step 2 are not shown). All results were replicated, except the N200 result at Step 1, in Two-Way ANOVA (Appendix F, Table A6). Importantly, the factor “participant” should be kept in mind (Appendix F, Table A6).

##### Target Irregularities Versus Nontarget Irregularities

LD and GS did not differ at Step 1 or Step 2. The separate Step 2_nonr_ and Step 2_resp_ also did not differ from each other. A difference between them was found only at Step 3 for MMN (0.72–0.16 s, *p* = 0.024). No statistically significant difference for later effects between pairs was found (Figure 5E–H; data for Step 2 are not shown).

GD and LS did not differ at Step 1 and Step 2 either as well as at Step 2_nonr_ and Step 2_resp_ separately. A difference between them was found only at Step 3 for late broad posterior positivity (0.196–0.696 s, *p* = 0.016). No statistically significant difference in early effects was found (Figure 5M–P; data for Step 2 are not shown).

## 3. Discussion

Our results suggest that even during a forced awakening from sleep, it takes tens of seconds to reach high levels of consciousness and regain full awareness and responsiveness to external stimuli.

Based on visual analysis of EEG and behavioral data, the transition from sleep to wakefulness was divided into steps. According to the recovery of alpha-activity and MR, we identified three basic steps in all awakenings from both SWS and REM: (1) either delta or theta waves prevail in EEG, and MR is not registered; (2) alpha-rhythm recovered and predominate in EEG, and MR is absent or slowed down; and (3) MR is comparable to those in the waking state before sleep.

Forced awakenings from SWS and REM showed essential differences in the speed of awakening and probability of reaching high levels of wakefulness. The percent of full awakenings (when accurate and timely MR to target stimuli was obtained) was significantly lower when woken during SWS; additionally, both the recovery of the alpha-rhythm and the restoration of the MR came later. However, there are some common patterns of awakening from SWS and REM. In particular, both during SWS and REM awakenings, first, alpha-activity was restored in the EEG, and only then MR to target stimuli recovered. Thus, our data show that the appearance of EEG predominating alpha-activity is not sufficient for organization of a MR. Moreover, after alpha appearance, it takes a surprisingly long time before MR is restored: more than 20 s elapses between the alpha recovery and the moment when MR becomes accurate and timely (more than 25 s after SWS and more than 20 s after REM). At this point, our data are in line with the previous observation of Langford et al. [11], who showed an essential delay between alpha-rhythm onset and voluntary response both in NREM2 and REM. Why are the participants not answering during this period? Is it difficult to organize a response? Is the processing of stimuli impaired? Or are they not remembering what is required of them?

To answer these questions, we analyzed ERPs on local and global irregularities on consecutive steps of awakening. During awakening from SWS at Step 1, when delta and theta rhythms predominated in EEG, no cognitive components of ERPs or MR to target trials were found. This result may be due to disrupted cortico-cortical connectivity, which was shown to be impaired when slow delta persists in EEG [18,39,40,41,42]. Cortico-cortical connectivity appears to be a primary mechanism of consciousness [17], and it was shown that, for its maintenance, alpha-activity is needed [18,19,43]. At Step 2_resp_, when alpha-rhythm recovered in EEG, but MR was slowed down, we found for the first time P3a to local irregularity. However, no significant cognitive components were found to global irregularity. The processing of global violations implies actively maintaining attention across several seconds [27] and requires the involvement of higher-order networks [28,31,32,35] that can be disrupted during the first seconds of awakening. Thus, it can be assumed that at Step 2, albeit alpha-rhythm has recovered, the neuronal processes are still insufficient for higher-level attentional processing.

Local irregularity is a short-time scale violation (the appearance of one deviant sound), so it is easier to process than a global one (the appearance of a deviant pattern of sounds); this also confirms the shorter reaction time. However, at Step 2_resp_, although the P3a to a violation of local regularity recovered, a comparison of the LD response to nontarget GS shows that they did not differ. This result could mean that there was no difference between trials where the same sound was the target and those where it was the nontarget. This brings us to the conclusion that neuronal processes underlying Step 2_resp_ of awakening from SWS were sufficient for activation of low-order pre-attentive auditory processing [44,45] but not sufficient for significance assessment, even in trials with short-time scale violation. The significance assessment requires a synthesis of sensory inflow with memory traces [46]. It was shown that features of consciousness, such as memory retrieval [3], voluntary control of actions [47], and significance assessment [28,31,32], require activation of a large-scale fronto-parietal system [48,49]. Our data suggest that these features fully recover only at Step 3 of awakening when the P300-like ERP component first emerges on target stimuli together with accurate and timely MR.

During awakening from REM, alpha-activity and MR on target stimuli recover earlier than during awakening from SWS. Analysis of ERPs showed that lower-order information processing also recovers faster; even at Step 1 of awakening, when theta activity prevails in EEG, and MR is not registered, we observed P3a for local irregularity. Similarly, for sequences with global irregularity, at Step 2, we found P3a in response to high sound in GS trials, containing the same stimuli as the LD trials in sequences with local irregularity. However, as during SWS awakening, we did not find any significant responses to GD at the first steps, and there was no difference in pairs LD vs. GS and GD vs. LS. P3b and P300 responses to target local and global deviants were also found only at Step 3 when MR became accurate and timely.

Thus, we can conclude that at the early steps of awakening, single deviant sounds are perceived by the brain, as evidenced by the appearance of the P3a component. However, at the same time, the ERPs to target stimuli do not differ from those to nontarget, i.e., the brain still cannot differentiate a target stimulus from an untargeted one. So, regarding the cause of the delay between alpha-rhythm onset (i.e. EEG-awakening) and voluntary response recovery (i.e. behavioral awakening), our data point to an impaired ability to assess the significance of the stimulus rather than the inability to move. Somewhat unexpected was that we did not observe the earlier recovery of cognitive components of ERPs and MR (either delayed or timely) to local irregularities than to global ones. This suggests that the main problem in the first seconds of awakening is not the impaired processing of complex auditory stimuli, but rather it is difficult to recollect what it is required to do.

Our results raise the question of what the role of alpha-rhythm in the recovery of consciousness is. Since local processing persists before alpha appearance when woken during REM, but, on the other hand, it can be disrupted even after alpha recovery when waking during SWS, is alpha-activity a reliable criterion of awakening?

During awakening from SWS, all markers of consciousness recovery (lower-order pre-attentive and high-level conscious processing of stimuli and MR to them) appear later than during awakening from REM. This difference may occur because forced awakening from SWS is closely related to the term “sleep inertia” [50,51]. During the transition from SWS to wakefulness, regional activation/deactivation patterns that mediate conscious perception recover more slowly than during that from REM (for metabolic changes [52]; for changes in EEG patterns of activation [53,54]; for latency and amplitude changes in ERPs [55,56,57]). This delay can cause a slower recovery of conscious response to target stimuli, as evidenced by data on worse behavioral performance after awakening from SWS compared to awakening from REM [58].

Faster recovery of pre-attentive processing during awakening from REM may also be explained by the fact that, during REM, cortico-cortical connectivity is preserved compared to SWS [21] but still differs from that in the waking state [59]. This difference may be why violation detection recovers faster than it does during awakening from SWS and P3a in response to local irregularity emergence even at Step 1. This may explain the particular importance of REM fragmentation in the pathogenesis of insomnia [60]: considering evidence showing increased micro- and macro-arousals during REM in insomnia patients [61], we may assume that faster emergence of consciousness from REM may lead to better memory consolidation and, ultimately, to an overestimation of the duration of awakenings. However, neither a higher level of activation nor a higher level of cortico-cortical connectivity during REM provides conditions for immediate recovery of high-level conscious processing of stimuli, so significant ERP components to global irregularity were not found until Step 3 when accurate and timely MR became fully restored.

Our data show that during awakening from both SWS and REM, the P300-like response to target stimuli, no matter simple (local irregularities) or complex (global irregularities), recovers only at Step 3 when the reaction accuracy becomes comparable to those registered in wakefulness. How exactly did the organization of neural activity change at the last step of awakening, when conscious awareness of external stimuli emerges? Unfortunately, we cannot answer this question within the framework of this paper. The answer requires further analysis of EEG spectral power and connectivity on consecutive steps of awakening.

## 4. Limitations

This study is the first of its kind on the recovery of conscious processing of external stimuli during the first seconds of awakening from natural sleep, and we tried to study this process both on individual and group levels. However, we should note some limitations of the study that can be further turned into research. The number of segments for ERP averaging is relatively small at Step 1 and also at Step 2 after dividing on Step 2_resp_ and Step 2_nonr_. However, this drawback is mostly connected to the peculiarity of the fast-changing awakening process and excessive movement activity at first steps, especially during awakening from SWS. In addition, importantly, we understand that because of design (20% of deviants vs. 80% of standards), a discrete variable (responses) occurs only on a minority number of trials in comparison to a continuous variable, such as alpha, which would be more likely to reappear sooner. Therefore, more experiments are needed to increase the statistics, or a more sensitive mathematical method of data analysis is needed (i.e., single-trial analysis). Moreover, in this work, we did not focus on parts of the nights during which participants were woken up, but they will be under investigation in future works regarding the control of circadian factors.

Additionally, we want to emphasize that waking from sleep is not consecutive and dogmatic, as in the progression through Step 1, Step 2, and Step 3. There are many more variations of the states between sleep and wakefulness: from no awakening at all to the absence of Step 2, partial awakenings, or fluctuations between states with periodically returning to sleep or previous steps. Therefore, the variety of these types of awakening may broaden our understanding of various degrees of consciousness and the mechanisms that underlie them.

In this work, we focused more on ERP and behavioral data. Therefore, in our further work, we will go a little deeper and focus on the spectral and connectivity analysis on consecutive steps of awakening. So, we will try to answer the question of how exactly the organization of neural activity changes, as first lower-order pre-attentive and then higher-order conscious auditory processing is restored, and to differentiate the necessary and sufficient processes mediating these levels of consciousness.

## 5. Conclusions

During awakening from sleep, first, alpha-activity restores in the EEG, and only after tens of seconds after alpha onset does the MR to target stimuli recover. Thus, during awakening, the appearance in EEG predominating alpha-activity is not sufficient either for conscious awareness of external stimuli or for generating a MR to them. This raises the question of whether the appearance of alpha-activity is a reliable criterion of awakening.

We found the P300-like response on target stimuli, both for local and global irregularities, only at a late stage of awakening when accurate and timely MR became fully restored. So, regarding the cause of the delay between an EEG-awakening and behavioral awakening, our data point to an impaired ability to assess the significance of the stimulus rather than the inability to move. There was no difference in the latency of MR recovery between local and global irregularities. Therefore, it seems that the main problem in the first seconds of awakening is not the impaired processing of complex auditory stimuli but rather the impaired memory retrieval.

SWS and REM differ in the speed of awakening: during awakening from REM, all successive steps of awakening emerge earlier. Obtained results may broaden our understanding of the pathophysiology of sleep disorders such as insomnia as well as conditions characterized by the dissociation between behavior and various aspects of consciousness.

## 6. Materials and Methods

### 6.1. Participants

Eight male volunteers (23 ± 3.02 years) without neurological or sleep disorders participated in the study. The eligibility criteria for the participants included the following: right-handed; aged 20–29 years; no history of hearing impairments, brain injury, neurological diseases, or sleep complaints; non-ongoing users of medications; no shift work; and no excessive daytime somnolence (i.e., ESS < 13). All participants were instructed to refrain from alcohol for 24 h and from caffeine (i.e., tea and coffee) for 6 h before the laboratory visit. The research protocols were compiled according to the Helsinki Declaration’s requirements, and the local ethical committee approved the study at the Institute of Higher Nervous Activity and Neurophysiology of the Russian Academy of Sciences. Before the first experiment, all participants were provided written informed consent, and after each experiment they received a monetary reward of 1000 RUB (13.67 USD).

### 6.2. Procedure

The week before the experiments, the volunteers were instructed to maintain a regular sleep-wake cycle with bedtimes between 23:00–01:00 h and wake-up times between 07:00–09:00 h and refrain from taking naps during the day. Compliance with these instructions was confirmed by accelerometric recordings (Xiaomi Mi Band). Volunteers were asked to complete a daily sleep diary and wear a wrist actigraph during the week before each experimental session. All participants had a regular sleep-wake cycle without jetlag in the 4 weeks before taking part in the experimental sessions, a habitual sleep duration, mean ± SD, 7:48 ± 0:35 (range: 7,5–9 h), bedtimes of 00:13 ± 0:21 (range: 00:00–01:00), and wake times of 08:10 ± 0:50 (range: 07:00–09:20). On the day before the first experimental session, the participants were asked to avoid drinks that could induce insomnia, such as coffee, tea, or cola.

**Figure A1:**
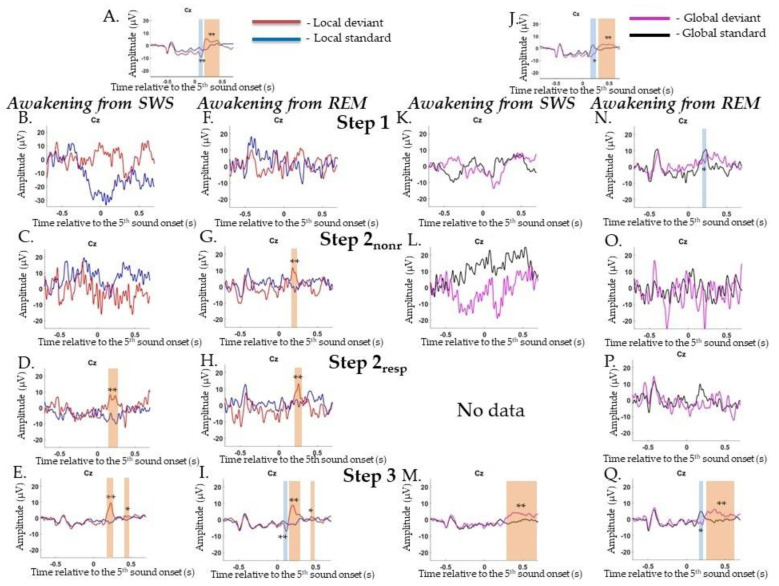
Event-related responses to violation of local regularity and global regularity in the sessions with awakening from slow-wave sleep and rapid eye movement sleep. SWS, slow-wave sleep; REM, rapid eye movement sleep; Step 1, period of awakening when delta or theta prevail in EEG, the motor response is absent; Step 2_nonr_, period of awakening when alpha prevails in EEG, the motor response is absent; Step 2_resp_, period of awakening when alpha prevails in EEG, the motor response is slow; Step 3, period of awakening when alpha prevails in EEG, the motor response is accurate and timely; s, second; red line, averaged local deviants; blue line, averaged local standards; magenta line, averaged global deviants; black line, averaged global standards; No data, no data went into analysis. (**A**) Local mismatch response in EEG in Wake_pre_ session. (**B**–**E**) The session with awakening from SWS for local irregularity. (**F**–**I**) The session with awakening from REM for local irregularity. (**J**) Global mismatch response in EEG in Wake_pre_ session. (**K**–**M**) The session with awakening from SWS for global irregularity. (**N**–**Q**) The session with awakening from REM for global irregularity. The blue-shaded region is a pool of negative clusters. The orange-shaded region is a pool of positive clusters. * *p* < 0.025, ** *p* < 0.01.

**Figure A2:**
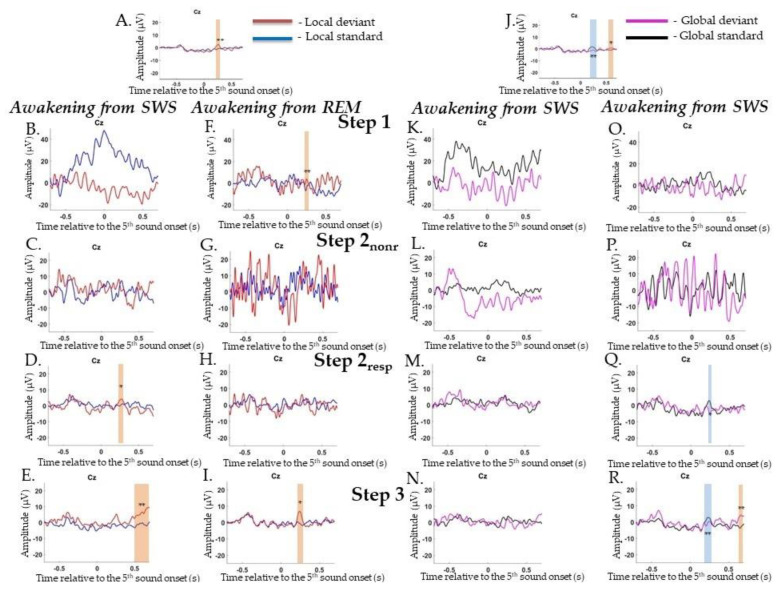
Event-related responses to violation of local regularity and global regularity in the sessions with awakening from slow-wave sleep and rapid eye movement sleep. SWS, slow-wave sleep; REM, rapid eye movement sleep; Step 1, period of awakening when delta or theta prevail in EEG, the motor response is absent; Step 2_nonr_, period of awakening when alpha prevails in EEG, the motor response is absent; Step 2_resp_, period of awakening when alpha prevails in EEG, the motor response is slow; Step 3, period of awakening when alpha prevails in EEG, the motor response is accurate and timely; s, second; red line, averaged local deviants; blue line, averaged local standards; magenta line, averaged global deviants; black line, averaged global standards; No data, no data got into analysis. (**A**) Local mismatch response in EEG in Wake_pre_ session. (**B**–**E**) The session with awakening from SWS for local irregularity. (**F**–**I**) The session with awakening from REM for local irregularity. (**J**) Global mismatch response in EEG in Wake_pre_ session. (**K**–**N**) The session with awakening from SWS for global irregularity. (**O**–**R**) The session with awakening from REM for global irregularity. The blue-shaded region is a pool of negative clusters. The orange-shaded region is a pool of positive clusters. * *p* < 0.025, ** *p* < 0.01.

Each volunteer participated in one adaptation night. In the pilot study, on volunteer participated, and we conducted 15 experimental sessions (ten sessions with awakenings from SWS—29 awakenings—and five sessions with awakenings from REM—20 awakenings) to obtain enough data for statistical analysis (see Appendix G, Figure A1). Then, we gained data from another participant to see whether the obtained effects could be replicated. He took part in 10 experimental sessions (five sessions with awakenings from SWS—16 awakenings—and five sessions with awakenings from REM—18 awakenings) (see Appendix H, Figure A2). Because the effects in these two participants were similar, we continued gaining data on other volunteers but with a smaller number of experiments (two sessions with awakenings from SWS and two sessions with awakenings from REM). Considering the improved effects in eight people, the united data are presented. In total, there were 77 awakenings from SWS (3 awakenings were excluded due to technical issues, and 12 awakenings were excluded because later they were scored as awakenings from N2) and 86 awakenings from REM.

The participants arrived at the research unit at 21:00 h for each experimental session. After the participants consumed a meal (21:30 h) standardized for all participants, they completed the Visual Analog Sleepiness Scale (VASS) and Stanford Sleepiness Scale (SSS). All tests were completed at 22:10 h. At 22:30 h, participants were prepared for nocturnal polysomnography. Electrodes were attached for registration of electroencephalography (EEG), electrooculography, and electrocardiography. At 22:40 h, the participants went to bed. In bed, they underwent a training session before sleep (Wake_pre_). They differentiated deviants in LGP (with the thumb, they clicked the button at a finger mouse ring when they heard a target pattern). Before sleep, the participants received the following instruction: “You are a fireman. Your task is to wake up as soon as possible when you hear an alarm (a sound 535 Hz, 100 dB intensity, 1-s duration) and start pressing the button when you hear a target pattern (either a pattern, in which the fifth sound is higher (for local irregularity), or the pattern, in which five sounds are the same (for global irregularity)). When you wake up, please, try not to move”. After that, the lights were turned off. The whole night, the participants were sleeping with the finger mouse ring on the right index finger. After every awakening, they were asked whether they had remembered the instruction (which irregularity was the target one); then, they were told which irregularity would be the target during the next awakening. The next day, at 07:00 h, the participants were woken up. Immediately after waking, while still in bed, they had a session after sleep (Wake_post_). In the room, they completed the VASS and SSS. After removing the electrodes, participants had breakfast.

### 6.3. Materials

#### 6.3.1. Test Instruments for Vigilance and Sleep Assessment

Before and after sleep, participants completed the sleepiness questionnaires: the Visual Analog Sleepiness Scale (VASS) and the Stanford Sleepiness Scale (SSS).

#### 6.3.2. Auditory Paradigms and Stimuli

We used the local-global paradigm (LGP) adapted from the study of Bekinschtein et al. [27]. The first four stimuli in the trial were identical, while the fifth stimulus was either the same or different. The local irregularity block contained 80% local standard (LS) SSSSS trials and 20% local deviant (LD) SSSSD trials; both appeared randomly. The global irregularity block contained 80% global standard (GS) SSSSD trials and 20% global deviant (GD) SSSSS trials. Both sounds were a harmonic tone composed of three sinusoidal partials of, e.g., 485, 970, and 1455 Hz and 515, 1030, and 1545 Hz. We were changing tones to avoid adaptation to them. The duration of the sounds was 50 ms (including 5 ms rise and fall times). Each sound in the trial sequence was separated by a 100 ms interstimulus interval (ISI), while the SOA between trials was 1400 ± 50 ms. The sounds were emitted by two loudspeakers located approximately 30 cm behind the participant’s head. In wakefulness, the counterbalanced presentation of GLP sounds consisted of two blocks (1 block with local irregularity and 1 block with global irregularity). A total of 280 standard and 72 deviant trials were presented in an 8 min 13 s block; the first 10 were standard patterns. During awakening, either local or global irregularity was presented (70 standard and 18 deviant trials were presented in a 2 min 04 s block). Sequences were presented in a random order in each experimental session to avoid automatized responses or reduced P300 amplitude. The presentation of stimuli sequences, synchronization with EEG recording, and registration of subjects’ responses were performed by Presentation 22.0 (Neurobehavioral Systems, Inc., Berkeley, CA, US).

#### 6.3.3. Data Acquisition and Polysomnography

Continuous EEG data were acquired by a digital 19-channel EEG amplifier Encepha-lan-EEGR-19/26 (Medicom, Taganrog, Russia), where 19 AgCl electrodes (Fp1, Fp2, F7, F3, Fz, F4, F8, T3, C3, Cz, C4, T4, T5, P3, Pz, P4, T6, O1, O2) were placed according to an extended international 10–20 system. An additional 4 electrodes were used for the acquisition of electrooculograms (EOGs). For recording vertical EOG, electrodes were placed 1 cm above and below the left eye. For recording horizontal EOG, electrodes were placed 1 cm lateral from the outer canthi of both eyes. Left and right mastoid electrodes served as reference channels for the monopolar design of EEG recording. Impedance was kept below 10 kΩ. A bandpass filter was set from 0.05 to 70 Hz, and the sampling rate was 250 Hz.

Polysomnograms were scored offline by two scorers who were blinded to the experimental conditions. Visual scoring of each 30 s epoch of PSG recording as awake, NREM stage NREM1, NREM2, or NREM3 (SWS), and REM was performed according to standard AASM criteria [20]. Interscorer reliability was >93%.

Total sleep time (TST), sleep period time (SPT), sleep onset latency (SOL), SWS and REM latencies, minutes spent in NREM1, NREM2, SWS, REM, wake, and sleep efficiency (SE) were analyzed. SPT was measured as the period beginning when the participant fell asleep and ending at the last wake-up, including the duration of awakenings if they occurred. SE was calculated as a percent value of TST referred to as SPT.

### 6.4. Preprocessing and Statistical Data Analyses

#### 6.4.1. Reaction Time

We considered reaction times (RTs) for the behavioral task before sleep, during awakenings, and after sleep for sequences with global and local irregularities separately. For Step 2_resp_, the reaction time was counted from the registered motor response (MR) to a deviant pattern even when MR was separated from the deviant pattern by one-two standard patterns. For Step 3, the response was considered accurate and timely if it did not exceed 550 milliseconds.

#### 6.4.2. Event-Related Potentials

Data were analyzed in BrainVision Analyzer 2.1 (Brain Products GmbH, Gilching, Germany). First, a high pass filter of 0.5 Hz, a low-pass filter at 30 Hz, and a notch filter at 50 Hz were applied. After that, we visually inspected and manually cleared continuous EEG from periods with muscle and motion artifacts. To remove eye-movement and blink-related EEG artifacts, independent component analysis (ICA) only in those awakenings where participants opened their eyes was conducted. In the LGP, epochs were segmented from 700 ms before to 700 ms after onset of the fifth sound in the sound trial. We used a baseline correction −700 to −600 ms window before the presentation of the fifth tone. Additionally, we performed an automatic artifact rejection of EEG epochs. We defined the following criteria for artifact rejection: a voltage step of more than 100 µV/ms, a voltage difference of less than −100 µV or more than 100 µV, and a maximum voltage difference of more than 100 µV within 100 ms intervals.

#### 6.4.3. Statistical Analysis

The data analyses were performed using Statistica 10 software (Stat Soft. Inc., Tulsa, OK, USA). All the data were evaluated for a normal distribution using the Shapiro–Wilks W test. Statistical significance was set at *p* < 0.05. The difference within sessions was subjected to the Wilcoxon signed-rank test and the difference between sessions and irregularities to the Mann–Whitney U test. Fisher’s exact test was conducted to assess the differences in the types of awakenings and parts of the nights between experimental sessions. A statistical comparison of ERPs was performed by cluster permutation tests corrected for multiple comparisons over time (Fieldtrip, Monte Carlo method, 500 permutations at 19 electrodes, alpha level for the two-sided permutation t tests = 0.025, corrected cluster forming threshold = 0.05 on a minimum of 2 neighboring electrodes) in the window between 0–700 ms after the fifth stimulus onset in the trial (FieldTrip toolbox running under MATLAB 2018b platform, The MathWorks, Inc., Natick, MA, USA) [62]. If there were no differences were found in the 0–700 ms window, additionally, the difference be-tween deviants and standards was checked in the window where they were visually differed.

Considering different number of sessions and awakenings in our participants, an additional control was conducted. The data were subjected to Two-Way ANOVA (MATLAB 2018b, The MathWorks, Inc., Natick, MA, USA).

## Figures and Tables

**Table 1:** Between sessions comparison of latency of each Step of awakening.

	Step 1			Step 2_nonr_			Step 2_resp_			Step 3		
	SWS ^a^ (*N* = 39)	REM ^a^ (*N* = 55)	*p*	SWS ^a^ (*N* = 35)	REM ^a^ (*N* = 52)	*p*	SWS ^a^ (*N* = 33)	REM ^a^ (*N* = 49)	*p*	SWS ^a^ (*N* = 38)	*REM ^a^* *(N = 54)*	*p*
Latency, s	0	0	1	15.24 ± 19.82	2.42 ± 2.35	**<0.001**	21.52 ± 13.55	8.41 ± 7.05	**<0.001**	40.22 ± 20.87	22.72 ± 14.99	**<0.001**

Note. Data are mean values ± standard deviation. SWS, slow-wave sleep; REM, rapid eye movement sleep; *N*, the number of awakenings. Step 1, period of awakening when delta or theta prevail in EEG, the motor response is absent; Step 2_nonr_, period of awakening when alpha prevails in EEG, the motor response is absent; Step 2_resp_, period of awakening when alpha prevails in EEG, the motor response is slow; Step 3, period of awakening when alpha prevails in EEG, the motor response is accurate and timely. Latency, time from the alarm sound to the beginning of the Step. Significant differences are highlighted in bold. *p*-values for each Step between sessions differences are derived from the Mann–Whitney U test. ^a^, to compare the speed of awakenings from SWS and REM, we calculated the latencies of Steps of only full awakenings when the participant reached Step 3. In addition, we excluded from analysis awakenings which contained returning to sleep or previous steps, and in some awakenings, we excluded steps with an excessive movement activity.

**Table 2:** Between sessions comparison of latency of MR recovery and reaction time on local or global irregularities in a waking state and during awakenings.

	Awakening from SWS			Awakening from REM			Awakening SWS + REM		
	Local ^a^	Global ^a^	*p*	Local ^a^	Global ^a^	*p*	Local ^a^	Global ^a^	*p*
Latency									
Step 2_resp,_ sec	21.98 ± 14.14 (*N* = 17)	21.02 ± 13.34 (*N* = 16)	0.871	6.9 ± 5.19 (*N* = 22)	9.63 ± 8.16 (*N* = 27)	0.131	13.47 ± 12.51 (*N* = 39)	13.87 ± 11.66 (*N* = 43)	0.417
Step 3, sec	37.56 ± 17.63 (*N* = 20)	43.18 ± 24.15 (*N* = 18)	0.693	19.43 ± 12.82 (*N* = 28)	26.26 ± 16.54 (*N* = 26)	0.206	26.98 ± 17.37 (*N* = 48)	33.18 ± 21.46 (*N* = 44)	0.282
Reaction time									
Wake_pre_, ms	380.07 ± 79.83 (*n* = 11)	449.29 ± 62.23 (*n* = 12)	**0.029**	378.19 ± 76.73 (*n* = 19)	452.93 ± 86.79 (*n* = 19)	**0.007**	378.88 ± 76.5 (*n* = 30)	451.52 ± 77.09 (*n* = 31)	**<0.001**
Step 2_resp_, ms	768.78 ± 442.14 (*N* = 16)	807.87 ± 3511.45 (*N* = 16)	0.72	786.93 ± 182.27 (*N* = 17)	941.54 ± 358.78 (*N* = 25)	0.148	778.13 ± 329.13 (*N* = 33)	889.38 ± 357.65 (*N* = 41)	0.217
Step 3, ms	418.89 ± 64.48 (*N* = 20)	475.29 ± 58.43 (*N* = 18)	**0.007**	423.42 ± 61.33 (*N* = 28)	458.58 ± 58.58 (*N* = 26)	**0.043**	421.53 ± 62.02 (*N* = 48)	465.41 ± 58.43 (*N* = 44)	**<0.001**
Wake_post_, ms	392.34 ± 90.08 (*n* = 11)	468.77 ± 58.95 (*n* = 9)	**0.04**	394.57 ± 108.76 (*n* = 20)	449.78 ± 65.44 (*n* = 19)	**0.02**	393.78 ± 100.98 (*n* = 31)	455.88 ± 62.98 (*n* = 28)	**0.002**

Note. Data are mean values ± standard deviation. SWS, slow-wave sleep; REM, rapid eye movement sleep; latency, time from the alarm sound to the beginning of the Step; *n*, the number of sessions; *N*, the number of awakenings; Wake_pre_, performance before sleep; Wake_post_, performance after sleep. Step 2_resp_, period of awakening when alpha prevails in EEG, the motor response is slow; Step 3, period of awakening when alpha prevails in EEG, the motor response is accurate and timely. Significant differences are highlighted in bold. The difference between local and global irregularities separately for each session of awakening and for united data of SWS and REM in a waking state and for Step 2_resp_ and Step 3 was analyzed. *p*-values for between irregularities differences are derived from the Mann–Whitney U test. ^a^, to compare the reaction times and latencies of MR recovery, we analyzed data of only full awakenings when the participant reached Step 3. In addition, we excluded from analysis awakenings which contained returning to sleep or previous steps, and in some awakenings, we excluded steps with an excessive movement activity.

## Data Availability

The data presented in this study are available on request from the corresponding author.

## Appendix A

**Table A1:** Comparison of sleep characteristics in sessions with SWS and REM awakenings.

Sleep Parameter	Session with Awakening from SWS (*n* = 26) ^a^	Session with Awakening from REM (*n* = 22)	*p*
TST, min	387.83 ± 128.63	407.39 ± 43.78	0.583
SPT, min	438.74 ± 134.83	479.53 ± 52.6	0.789
NREM1, min	18.4 ± 9.6	19.53 ± 11.24	0.8
NREM2, min	185.67 ± 56.28	197.72 ± 42.75	0.662
SWS, min	89.24 ± 48.34	102.5 ± 24.24	0.242
REM, min	86.36 ± 41.46	75.78 ± 24.68	0.271
Wake, min	50.9 ± 36.25	72.14 ± 46.77	0.147
SOL, min	10.4 ± 5.73	20.36 ± 27.8	0.124
SWS latency, min	14.33 ± 18.81	13.92 ± 12.6	0.746
REM latency, min	90.19 ± 34.86	91.14 ± 48.66	0.622
SE, %	87.73 ± 7.93	85.42 ± 8.55	0.304

Note. Data are mean values ± standard deviation. SWS, slow-wave sleep; REM, rapid eye movement sleep; *n*, the number of sessions; TST, total sleep time; SPT, sleep period time; NREM, non-rapid eye movement sleep; NREM1, stage 1 of sleep; NREM2, stage 2 of sleep; SOL, sleep onset latency; SE, sleep efficiency. *p*-values for between sessions differences are derived from the Mann–Whitney U test. ^a^, there were 5 sessions of awakening from SWS, when the participants asked to take off the EEG cap after all awakenings.

## Appendix B

**Table A2:** Types of awakenings according to their completeness in sessions with SWS and REM awakenings.

	Session with Awakening from SWS (*N* = 77)	Session with Awakening from REM (*N* = 86)	*p*
No awakenings	29 (37.7)	17 (19.8)	0.023
Partial awakenings	20 (25.9)	15(17.4)	0.252
Full awakenings	28 (36.4)	54 (62.8)	0.001

Note. Data are number of types of awakenings, and percentage is in brackets. SWS, slow-wave sleep; REM, rapid eye movement sleep; *N*, the number of awakenings. *p*-values for between sessions differences are derived from the Fisher’s exact test, two-tailed. Significant differences are highlighted in bold.

## Appendix C

**Table A3:** Part of the night of awakening.

	Session with Awakening from SWS (*N* = 77)	Session with Awakening from REM (*N* = 86)	*p*
Part 1 (23:00–03:30)	48 (62.3)	30 (34.9)	<0.001
Part 2 (03:30–08:00)	29 (37.7)	56 (65.1)	

Note. Data are number of sessions, and percentage is in brackets. SWS, slow-wave sleep; REM, rapid eye movement sleep; *N*, the number of awakenings. *p*-values for between sessions differences are derived from the Fisher’s exact test, two-tailed. Significant differences are highlighted in bold.

## Appendix D

**Table A4:** Comparison of sleepiness assessment in Wake_pre_ and Wake_post_ in sessions with SWS and REM awakenings.

Parameter	Wake_pre_ in the Session with Awakening from SWS (*n* = 25) ^a^	*p* (SWS)	Wake_post_ in the Session with Awakening from SWS (*n* = 25) ^a^	Wake_pre_ in the Session with Awakening from REM (*n* = 22)	*p* (REM)	Wake_post_ in the Session with Awakening from REM (*n* = 22)	*p* (Wake_pre_ SWS vs. REM)	*p* (Wake_post_ SWS vs. REM)
VASS, mm	6.14 ± 1.85	**<0.001**	3.93 ± 1.46	5.69 ± 1.99	**0.002**	3.46 ± 1.45	0.332	0.277
SSS	4.08 ± 1.26	**0.01**	3.16 ± 1	4.14 ± 1.13	**<0.001**	2.77 ± 0.87	0.848	0.216

Note. Data are mean values ± standard deviation. SWS, slow-wave sleep; REM, rapid eye movement sleep; Wake_pre_, performance before sleep; Wake_post_, performance after sleep; *n*, the number of sessions; VASS, Vigilance Analog Sleepiness Scale in millimeters; SSS, Stanford Sleepiness Scale (score from 1 to 7). Significant differences are highlighted in bold. *p* (SWS) and *p* (REM), performance before and after sleep separately for two sessions of awakening was analyzed. *p*-values for within sessions differences are derived from the Wilcoxon’s matched pairs test. *p* (Wake_pre_ SWS vs. REM) and *p* (Wake_post_ SWS vs. REM), corresponding parameter in two sessions of awakening was analyzed. *p*-values for between sessions differences are derived from the Mann–Whitney U test. ^a^, there was 1 session of awakening from SWS, when the participant did not fill in the questionnaires before and after sleep.

## Appendix E

Considering the different number of sessions and awakenings in our participants, Two-Way ANOVA with two factors—“stimuli” (deviant or standard) and “participant” (eight volunteers) and “interaction” (stimuli × participant)—was conducted (MATLAB 2018b, The MathWorks, Inc., Natick, MA, USA). We analyzed the maximum of amplitude of local standards and local deviants of each ERP in previously detected windows, in FieldTrip, Matlab, for each participant’s awakening (for more details, 2.4.2—for SWS awakenings and 2.4.3—for REM awakenings in the article) at the Cz electrode. d.f.. The η^2^p, *p*-value, and F-value and d.f. error for the data, where the difference between standards and deviants was detected in FieldTrip, Matlab, are shown.

**Table A5:** Comparison of maximum amplitude difference between sounds for local irregularity.

Performance before and after Sleep														
		Deviants	Standards	Stimuli				Participant				Interaction		d.f. error
				d.f.	η^2^p	F	*p*	d.f.	η^2^p	F	*p*	d.f.	η^2^p	
Wake_pre_	MMN	3.26 ± 1.72	−0.85 ± 0.88	1	0.6	104.82	**<0.001**	7	0.3	4.19	**<0.001**	7	0.33	70
	P3a	7.28 ± 4.31	2.09 ± 1.22	1	0.53	79.43	**<0.001**	7	0.49	9.57	**<0.001**	7	0.27	
	P3b	8.36 ± 3.25	4.29 ± 1.74	1	0.57	93.39	**<0.001**	7	0.55	12.22	**<0.001**	7	0.19	
Wake_post_	MMN	−3.07 ± 1.85	−0.95 ± 0.61	1	0.49	63.24	**<0.001**	7	0.31	4.21	**<0.001**	7	0.2	66
	P3a	6.04 ± 3.72	1.35 ± 0.85	1	0.61	103.25	**<0.001**	7	0.57	12.31	**<0.001**	7	0.51	
	P3b	7.01 ± 3.41	3.69 ± 1.68	1	0.46	56.07	**<0.001**	7	0.6	14.12	**<0.001**	7	0.17	
**SWS**														
		**Deviants**	**Standards**	**Stimuli**				**Participant**				**Interaction**		**d.f. error**
				**d.f.**	**η^2^p**	**F**	***p***	**d.f.**	**η^2^p**	**F**	***p***	**d.f.**	**η^2^p**	
Step 1	P3a	-	-	-	-	-	-	-	-	-	-	-	-	-
Step 2	P3a	7.75 ± 7.63	1.65 ± 4.69	1	0.13	8.35	**0.005**	7	0.09	0.86	0.543	7	0.15	58
Step 2_nonr_	P3a	-	-	-	-	-	-	-	-	-	-	-	-	-
Step 2_resp_	P3a	10.42 ± 7.89	2.88 ± 3.7	1	0.29	15.35	**<0.001**	5	0.18	1.72	0.154	5	0.19	38
Step 3	MMN	-	-	-	-	-	-	-	-	-	-	-	-	−56
	P3a	9.93 ± 6.05	3.57 ± 2.74	1	0.15	9.49	**0.003**	6	0.13	1.36	0.248	6	0.22	
	P3b	10.54 ± 5.03	5.77 ± 2.23	1	0.25	18.67	**<0.001**	6	0.15	1.61	0.163	6	0.06	
**REM**														
		**Deviants**	**Standards**	**Stimuli**				**Participant**				**Interaction**		**d.f. error**
				**d.f.**	**η^2^p**	**F**	***p***	**d.f.**	**η^2^p**	**F**	***p***	**d.f.**	**η^2^p**	
Step 1	P3a	14.45 ± 8.36	1.51 ± 7.72	1	0.34	5.23	**0.045**	1	0.02	0.29	0.601	1	0.04	10
Step 2	P3a	11.56 ± 7.66	5.00 ± 5.37	1	0.13	5.95	**0.019**	7	0.35	3.03	**0.012**	7	0.29	40
Step 2_nonr_	P3a	13.97 ± 9.82	2.72 ± 5.45	1	0.22	2.8	0.125	5	0.34	1.04	0.447	5	0.32	10
Step 2_resp_	P3a	9.69 ± 7.73	4.77 ± 3.8	1	0.31	8.81	**0.008**	6	0.52	3.68	**0.013**	6	0.37	20
Step 3	MMN	−4.5 ± 3.78	−1.65 ± 2.36	1	0.2	10.27	**0.003**	5	0.35	4.48	**0.002**	5	0.08	42
	P3a	9.8 ± 5.6	3.16 ± 2.19	1	0.19	9.59	**0.004**	5	0.42	6.2	**<0.001**	5	0.46	
	P3b	4.92 ± 3.69	3.47 ± 2.28	1	0.12	5.57	**0.023**	5	0.22	2.37	0.056	5	0.11	

Note. Data are maximum amplitude in µV of detected ERPs in FieldTrip, Matlab and d.f.. η^2^p, *p*-value, and F-value and d.f. error from the Two-Way ANOVA with two factors (“stimuli” and “participant”) and “interaction” (stimuli x participant) for local irregularity. Wake_pre_, performance before sleep; Wake_post_, performance after sleep; SWS, slow-wave sleep; REM, rapid eye movement sleep; MMN, Mismatch negativity, -, no ERPs were detected in FieldTrip. Step 1, period of awakening when delta or theta prevail in EEG, the motor response is absent; Step 2, period of awakening when alpha prevails in EEG, the motor response is either absent or slow; Step 2_nonr_, period of awakening when alpha prevails in EEG, the motor response is absent; Step 2_resp_, period of awakening when alpha prevails in EEG, the motor response is slow; Step 3, period of awakening when alpha prevails in EEG, the motor response is accurate and timely. Significant differences are highlighted in bold.

## Appendix F

Considering the different number of sessions and awakenings in our participants, Two-Way ANOVA with two factors—“stimuli” (deviant or standard) and “participant” (eight volunteers) and “interaction” (stimuli × participant)—was conducted (MATLAB 2018b, The MathWorks, Inc., Natick, MA, USA). We analyzed the maximum of amplitude of global standards and global deviants of each ERP in previously detected windows in FieldTrip, Matlab, for each participant’s awakening (for more details, 2.4.2 – for SWS awakenings and 2.4.3 – for REM awakenings in the article) at the Cz electrode. d.f.. The η^2^p, *p*-value, and F-value and d.f. error for the data, where the difference between standards and deviants was detected in FieldTrip, Matlab, are shown.

**Table A6:** Comparison of maximum amplitude difference between sounds for global irregularity.

Performance before and after Sleep														
		**Deviants**	**Standards**	Stimuli				Participant				Interaction		d.f. error
				**d.f.**	η^2^p	F	*p*	d.f.	η^2^p	F	*p*	d.f.	η^2^p	
Wake_pre_	N200	−1.68 ± 1.45	−0.43 ± 1.22	1	0.16	13.77	**<0.001**	7	0.16	1.93	0.077	7	0.06	70
	P300	7.51 ± 3.05	4.87 ± 1.63	1	0.44	54.69	**<0.001**	7	0.61	15.91	**<0.001**	7	0.25	
Wake_post_	N200	−1.65 ± 1.68	−0.48 ± 1.65	1	0.18	14.16	**<0.001**	7	0.48	8.66	**<0.001**	7	0.09	66
	P300	7.45 ± 3.82	4.53 ± 1.82	1	0.34	34.71	**<0.001**	7	0.5	9.25	**<0.001**	7	0.2	
**SWS**														
		**Deviants**	**Standards**	**Stimuli**				**Participant**				**Intersection**		**d.f. error**
				**d.f.**	**η^2^p**	**F**	***p***	**d.f.**	**η^2^p**	**F**	***p***	**d.f.**	**η^2^p**	
Step 1	N200	-	-	-	-	-	-	-	-	-	-	-	-	-
Step 2	N200	-	-	-	**-**	**-**	-	-	-	-	-	-	-	-
Step2_nonr_	N200	-	-	-	-	-	-	-	-	-	-	-	-	-
Step2_resp_	N200	-	-	-	-	-	**-**	-	-	-	-	-	-	-
Step 3	N200	−3.64 ± 6.83	−0.4 ± 3.33	1	0.02	1.84	0.18	6	0.07	0.91	0.49	6	0.04	72
	P300	11.69 ± 5.21	6.1 ± 2.75	1	0.23	22.07	**<0.001**	6	0.23	3.57	**0.004**	6	0.09	
**REM**														
		**Deviants**	**Standards**	**Stimuli**				**Participant**				**Interaction**		**d.f. error**
				**d.f.**	**η^2^p**	**F**	***p***	**d.f.**	**η^2^p**	**F**	***p***	**d.f.**	**η^2^p**	
Step 1	N200	−3.4 ± 8.86	4.91 ± 10.65	1	0.17	3.65	0.72	3	0.25	1.97	0.155	3	0.08	18
Step 2	N200	−6.91 ± 5.62	−1.95 ± 4.55	1	0.24	16.61	**<0.001**	7	0.17	1.56	0.168	7	0.1	52
Step2_nonr_	N200	−3.52 ± 9.29	1.27 ± 5.46	1	0.52	6.39	**0.045**	5	0.74	3.57	0.077	5	0.57	6
Step2_resp_	N200	−5.39 ± 4.55	−0.71 ± 3.64	1	0.29	14.01	**<0.001**	7	0.12	0.64	0.722	7	0.18	34
Step 3	N200	−3.06 ± 2.33	−0.73 ± 1.98	1	0.19	10.78	**0.002**	6	0.24	2.46	**0.038**	6	0.06	46
	P300	10.12 ± 3.13	5.95 ± 2.03	1	0.39	29.84	**<0.001**	6	0.21	2.02	0.083	6	0.08	

Note. Data are maximum amplitude in µV of detected ERPs in FieldTrip and d.f.. η^2^p, *p*-value, and F-value and d.f. error from the Two-Way ANOVA with two factors (“stimuli” and “participant”) and “interaction” (stimuli x participant) for global irregularity. Wake_pre_, performance before sleep; Wake_post_, performance after sleep; SWS, slow-wave sleep; REM, rapid eye movement sleep; -, no ERPs were detected in FieldTrip. Step 1, period of awakening when delta or theta prevail in EEG, the motor response is absent; Step 2, period of awakening when alpha prevails in EEG, the motor response is either absent or slow; Step 2_nonr_, period of awakening when alpha prevails in EEG, the motor response is absent; Step 2_resp_, period of awakening when alpha prevails in EEG, the motor response is slow; Step 3, period of awakening when alpha prevails in EEG, the motor response is accurate and timely. Significant differences are highlighted in bold.

## Appendix G

In the pilot study, one volunteer took part in 15 experimental sessions (ten sessions with awakenings from SWS—29 awakenings—and five sessions with awakenings from REM—20 awakenings).

### Appendix G.1. Local Irregularities

ERPs to local irregularities were analyzed by comparing responses to LD and LS as a contrast termed the local effect (LD-LS). In the Wake_pre_ session, a significant local effect was found for frontocentral MMN (0.72–0.128 s, *p* = 0.002), followed by the P3a/P3b complex (0.152–0.496 s, *p* = 0.002) (Figure A1A). In the Wake_post_ session, a similar result for local effects was found: MMN (0.68–0.132 s, *p* = 0.002), which was followed by broad positive complex P3a/P3b (0.152–0.532 s, *p* = 0.002) (data are not shown).

During awakening from SWS, P3a was found at Step 2_resp_ (0.212–0.276 s, *p* = 0.002) and the P3a/b complex at Step 3 (0.168–0.28 s, *p* = 0.002 and 0.388–0.5 s, *p* = 0.018) (Figure A1B–E), while during awakening from REM, P3a was found at all Steps, except Step 1, (0.164–0.208 s, *p* = 0.002, 0.2–0.208 s, *p* = 0.002, and 0.148–0.296 s, *p* = 0.002). At Step 3 also, MMN and P3b were detected (0.092–0.128 s, *p* = 0.002 and 0.404–0.476 s, *p* = 0.012, respectively) (Figure A1F–I).

### Appendix G.2. Global Irregularities

ERPs to violations of global regularities were analyzed by comparing responses to GD and GS. In the Wake_pre_ session, a significant global effect (GD–GS) was found for N200 (0.136–0.26 s, *p* = 0.02), followed by broad centroparietal P300 (0.256–0.664 s, *p* = 0.002) (Figure A1J). In the Wake_post_ session, a similar result for the global effect was found: N200 (0.14–0.232 s, *p* = 0.006), which was followed by broad centroparietal P300 (0.252–0.64 s, *p* = 0.002) (data are not shown).

During awakening from SWS, P300 was registered at Step 3 (0.268–0.696 s, *p* = 0.002) (Figure A1K–M), while during awakening from REM, N200 was registered at Step 1 and Step 3 (0.172–0.232 s, *p* = 0.012 and 0.14–0.228 s, *p* = 0.01, respectively), while P300 was detected only at Step 3 (0.248–0.608 s, *p* = 0.002) (Figure A1N–Q).

## Appendix H

The participant took part in 10 experimental sessions (five sessions with awakenings from SWS—16 awakenings—and five sessions with awakenings from REM—18 awakenings).

### Appendix H.1. Local Irregularities

ERPs to local irregularities were analyzed by comparing responses to LD and LS as a contrast termed the local effect (LD–LS). In the Wake_pre_ session, a significant local effect was found for the P3a/P3b complex (0.176–0.308 s, *p* = 0.002) (Figure A2A). In the Wake_post_ session, a similar result for local effects was found: positive complex P3a/P3b (0.204–0.312 s, *p* = 0.01) (data are not shown).

During awakening from SWS, P3a was found at Step 2_resp_ (0.232–0.272 s, *p* = 0.014) and P3b at Step 3 (0.58–0.696 s, *p* = 0.002) (Figure A2B–E), while during awakening from REM, P3a was found at all Steps 1 and Step 3 (0.232–0.236 s, *p* = 0.002 and 0.172–0.284 s, *p* = 0.014, respectively) (Figure A2F–I).

### Appendix H.2. Global Irregularities

ERPs to violations of global regularities were analyzed by comparing responses to GD and GS. In the Wake_pre_ session, a significant global effect (GD-GS) was found for N200 (0.112–0.296 s, *p* = 0.002), followed by late centroparietal P300 (0.524–0.636 s, *p* = 0.012) (Figure A2J). In the Wake_post_ session, a similar result for the global effect was found: N200 (0.148–0.316 s, *p* = 0.002), which was followed by late centroparietal P300 (0.608–0.688 s, *p* = 0.012) (data are not shown).

During awakening from SWS, no effects were found (Figure A2K–N), while during awakening from REM, N200 was registered at Step 2_resp_ and Step 3 (0.76–0.26 s, *p* = 0.01 and 0.152–0.252 s, *p* = 0.002, respectively), while late P300 was detected only at Step 3 (0.624–0.684 s, *p* = 0.002) (Figure A2O–R).
